# A DCM study of spectral asymmetries in feedforward and feedback connections between visual areas V1 and V4 in the monkey

**DOI:** 10.1016/j.neuroimage.2014.12.081

**Published:** 2015-03

**Authors:** A.M. Bastos, V. Litvak, R. Moran, C.A. Bosman, P. Fries, K.J. Friston

**Affiliations:** a Ernst Strüngmann Institute (ESI) in Cooperation with Max Planck Society, Deutschordenstraße 46, Frankfurt 60528, Germany; b Center for Neuroscience and Center for Mind and Brain, University of California, Davis, Davis, CA 95618, USA; c The Wellcome Trust Centre for Neuroimaging, University College London, Queen Square, London WC1N 3BG, UK; d Donders Institute for Brain, Cognition and Behaviour, Radboud University Nijmegen, Kapittelweg 29, Nijmegen 6535 EN, Netherlands; e Cognitive and Systems Neuroscience Group, Center for Neuroscience, Swammerdam Institute for Life Sciences, University of Amsterdam, Amsterdam 1098 XH, Netherlands

**Keywords:** Neuronal, Connectivity, Computation, Dynamic causal modeling, Synchronization coherence, Transfer functions, Gamma oscillations, Beta oscillations

## Abstract

This paper reports a dynamic causal modeling study of electrocorticographic (ECoG) data that addresses functional asymmetries between forward and backward connections in the visual cortical hierarchy. Specifically, we ask whether forward connections employ gamma-band frequencies, while backward connections preferentially use lower (beta-band) frequencies. We addressed this question by modeling empirical cross spectra using a neural mass model equipped with superficial and deep pyramidal cell populations—that model the source of forward and backward connections, respectively. This enabled us to reconstruct the transfer functions and associated spectra of specific subpopulations within cortical sources. We first established that Bayesian model comparison was able to discriminate between forward and backward connections, defined in terms of their cells of origin. We then confirmed that model selection was able to identify extrastriate (V4) sources as being hierarchically higher than early visual (V1) sources. Finally, an examination of the auto spectra and transfer functions associated with superficial and deep pyramidal cells confirmed that forward connections employed predominantly higher (gamma) frequencies, while backward connections were mediated by lower (alpha/beta) frequencies. We discuss these findings in relation to current views about alpha, beta, and gamma oscillations and predictive coding in the brain.

## Highlights

• We briefly review evidence for canonical microcircuits (CMC) and predictive coding.
• This evidence is incorporated into a novel Dynamic Causal Model (DCM).
• We model observed cross-spectral densities from monkey visual cortex (V1 and V4).
• We establish the face and predictive validity of this new DCM.
• Gamma rhythms subserve feedforward, and alpha/beta rhythms feedback influences.

## Introduction

This paper is about the asymmetries in effective connectivity among different levels of the visual cortical hierarchy. These asymmetries were quantified in terms of the spectral characteristics of sources, as measured with electrocorticographic (ECoG) local field potential (LFP) data from an awake-behaving monkey performing a visuospatial attention task. We used dynamic causal modeling to assign underlying neuronal activity to specific cell populations elaborating forward and backward connections among cortical areas. This enabled us to estimate the frequencies conveying forward and backward influences between sources at different hierarchical levels. In brief, we confirmed that forward connections are mediated by gamma frequencies, while backward connections appear to be conveyed by alpha/beta frequencies. These results rest upon two recent developments in the modeling of electrophysiological data: the first is the introduction of dynamic causal modeling for complex data, such as the complex cross spectra summarizing dependencies among recordings from different sites (Friston et al., 2012). The second development is the introduction of a neural mass model (based on a canonical microcircuit) that distinguishes between cell populations that give rise to forward and backward extrinsic connections. This model has been motivated from a theoretical perspective of predictive coding in Bastos et al. (2012). In addition, empirical evidence for a dissociation between gamma and beta in feedforward and feedback transmission in the visual system was recently demonstrated by Bastos et al. (2011, 2015). Given this theoretical and empirical motivation, we use dynamic causal modeling of empirical cross spectra to address, specifically, spectral differences between forward and backward connections and their underlying generative mechanisms.

This paper comprises four sections. The first section briefly reviews the empirical evidence for dissociations in the neuroanatomy, physiology, function, and frequency content of forward and backward message passing, and how these dissociations may be understood in terms of neuronal computations and distributed processing during perceptual inference. The second section then considers the more pragmatic issue of how to quantify asymmetries using mesoscopic and macroscopic electrophysiological measurements. This section constitutes a brief review of the empirical and theoretical motivation for the canonical microcircuit model used in the subsequent section for dynamic causal modeling. We then briefly review dynamic causal modeling, with a special focus on models of cross-spectral densities acquired under steady-state assumptions. The final section presents an analysis of empirical data that first establishes the face validity and the predictive validity of the model and then presents our results in terms of population-specific spectral behavior and directed connectivity in terms of transfer functions. We conclude with a discussion of these results in the light of current theories about inter-areal communication, oscillations, and message passing in the brain.

### Functional asymmetries in hierarchical connections

The importance of asymmetries between forward and backward connections has been established for several decades and yet the *in vivo* electrophysiological evidence for systematic differences has until recently remained somewhat indirect. Perhaps the most well-known asymmetry between feedforward and feedback connections was established by a series of seminal tract tracing studies (e.g., Rockland et al., 1979) reviewed by Felleman and Van Essen (1991). In this review, the authors examined patterns of anterograde and retrograde anatomical tracing studies made in several different areas of the macaque visual cortex and concluded that three canonical patterns of anatomical connectivity emerged across the many areas studied, which they termed feedforward, feedback, and lateral connections (Felleman and Van Essen, 1991). Feedforward connections canonically derived from the superficial pyramidal cells of the source area and targeted the granular layer of the recipient area, while feedback connections derived from the infragranular layers of the source area and terminated outside the granular layer of the recipient area. This observation led these authors to propose a hierarchical model of cortical processing organized into approximately ten levels, starting with area V1 at the bottom of the visual (cortical) hierarchy. This pattern of connectivity has recently been extended, with the observation that not all feedforward connections derive strictly from the supragranular layers. Instead, it appears that the ratio of projection neurons located in supragranular layer to projection neurons located in infragranular layers can be used as a rough marker for how stereotypically feedforward or feedback a given connection is—areas that are nearby to one another in the hierarchy will have a weaker supra—to infragranular asymmetry compared to areas that are separated by multiple hierarchical levels (Barone et al., 2000; Markov et al., 2013; Vezoli, 2004).

Physiologically, there is a variety of evidence for asymmetries in the functional characteristics of feedforward versus feedback projections. These asymmetries are clearest in the first-order thalamic nuclei such as the lateral geniculate nucleus (LGN), whose afferents can be separated into two classes—feedforward input from the retina and feedback from layer 6 of the first visual cortical area. These connections differ from each other in several ways: feedforward connections display strong initial EPSPs (excitatory postsynaptic potentials), use exclusively ionotropic glutamate receptors, and have depressing synapses to paired-pulse stimulation (Sherman and Guillery, 1998). Feedback connections terminate on the distal part of the dendritic arbor, evoke weaker EPSPs, are more modulatory in the sense that they employ both ionotropic and metabotropic synaptic components, and show paired-pulse facilitation (Sherman and Guillery, 2011). In addition, geniculocortical feedforward and corticogeniculate feedback functional connectivities have recently been shown to be asymmetric in the frequency domain: beta-band frequencies signaled in the feedforward (geniculocortical) direction and alpha-band frequencies signaled in the feedback (corticogeniculate) direction (Bastos et al., 2014a). One possible explanation for this dissociation of the faster frequency for feedforward communication and the slower frequency for feedback communication is that it may be due to the differences in synaptic physiology of the two directions.

In contrast to the LGN afferents, the evidence about the synaptic physiology of corticocortical connections is much more mixed. Two recent studies that examined the synaptic characteristics between mouse V1–V2 and A1–A2 found essentially no evidence for asymmetries in any of the properties that were previously discovered to discriminate feedforward and feedback connections at the level of the LGN (Covic and Sherman, 2011; De Pasquale and Sherman, 2011). At the level of single neurons, it is known that forward connections between V1 and higher cortical areas establish the basic receptive field characteristics of those higher areas because when V1 is experimentally cooled or lesioned to silence its activity, areas V2, V3, V3A, V4, and MT are either strongly reduced in their activity or activity is completely abolished (Girard et al., 1991, 1992; Girard and Bullier, 1989). This is consistent with a strong, driving role for the feedforward connections. In contrast, when the feedback connections are silenced, activity in earlier cortical areas appears to be only weakly affected, and the sign of the effect appears to depend on whether the extraclassical receptive field is stimulated or not (Bullier et al., 1996; Hupé et al., 1998). This indicates that feedback connections are more modulatory or nonlinear and may interact with activity in earlier areas in a complex way. A nonlinear, modulatory role for corticocortical feedback is also consistent with an early neuroimaging study that modeled fMRI responses to visual stimulation, and found that feedback connections between V2 and V1 were more modulatory in relation to the feedforward connection from V1 to V2 (Friston et al., 1995).

Another dissociation between feedforward and feedback connections is their valence (functionally excitatory or inhibitory). Feedforward connections are thought to produce the main excitatory drive to neurons in the visual system, while feedback connections have been associated with contextual processing that can often inhibit neuronal activity of earlier areas (for example, extraclassical receptive field effects). Although extrinsic, i.e., inter-areal, connections in the cortex are often to be exclusively excitatory (but see Melzer et al., 2012), an effective (polysynaptic) inhibitory effect could be mediated through several distinct corticocortical pathways, such as synaptic feedback termination in layer 1 and layer 6 (reviewed in Bastos et al., 2012). Corticocortical feedback connections terminate heavily in these layers (Shipp, 2007), and both layers appear to have an inhibitory influence on pyramidal cells in layers 2 to 5, presumably mediated by intrinsic, i.e., local, inhibitory interneurons (Meyer et al., 2011; Olsen et al., 2012; Shlosberg et al., 2006). This hypothesized inhibitory role for corticocortical feedback is consistent with a large literature in the neuroimaging field that has established that when neuronal responses are more predictable, neural activity in earlier areas tends to decrease, consistent with a predictive role for feedback connections (Alink et al., 2010; Garrido et al., 2009; Summerfield et al., 2008, 2011). Furthermore, a study using dynamic causal modeling (DCM) for induced responses measured with MEG documented a greater suppressive effect of feedback compared to feedforward connections, and this suppressive effect was specific to higher frequencies in higher cortical areas suppressing lower frequencies of lower cortical areas (Chen et al., 2009). The authors interpreted this (somewhat unexpected) result along the following lines: “Heuristically, this means that gamma activity in low-level areas induces slower dynamics at higher cortical levels as prediction error is accumulated for perceptual synthesis. The concomitant high-level gamma activity (due to intrinsic nonlinear coupling) then accelerates the decay of evoked responses in the lower level that are manifest at, the population level, as damped alpha oscillations” (Chen et al., 2009, p461). Importantly, (Chen et al., 2009) modeled induced responses phenomenologically. In the current paper, we build on these findings by modeling the neuronal dynamics that give rise to feedforward and feedback effects such as those reported by Chen et al., 2009. In other words, we try to account for the basic phenomena (asymmetric spectral coupling) in terms of biophysically plausible neuronal processes. Note that the current application of DCM does not require fluctuations in spectral responses; it operates directly on the relative expression of different frequencies in the cross spectra. In contrast, DCM for induced responses (e.g., Chen et al., 2009) models time-dependent changes in cross spectra induced by a stimulus.

This points to another emerging dissociation between forward and backward connections: their frequency content (Wang, 2010). Several lines of evidence now suggest that forward connections prefer gamma, or generally higher, frequencies while backward connections prefer alpha/beta, or generally lower, frequencies (Bastos et al., 2014a, 2015; Bollimunta et al., 2011; Bosman et al., 2012; Buschman and Miller, 2007; Fontolan et al., 2014; Maier et al., 2010; Roopun et al., 2006; Van Kerkoerle et al., 2014; Von Stein et al., 2000). Buffalo et al. (2011) recorded in the superficial and deep layers of monkey visual areas V1, V2, and V4. They found that single units in the superficial layers (the layers sending feedforward projections) showed clear, oscillatory spike-field coherence in the gamma range, while units in the deep layers (the layers sending feedback connections) lacked this gamma coherence and instead were coherent with the LFP in the alpha/beta range. Consistent with this asymmetry, Bosman et al. (2012) showed that between visual areas V1 and V4, gamma oscillations were much stronger in the feedforward as compared to the feedback direction. Furthermore, Bastos et al. (2015) found a dissociation between forward and backward connections across 28 pairs of areas in the visual system: Granger-causal influences in the gamma-band were consistently stronger in the feedforward direction, whereas in the majority of the connections, Granger-causal influence in the beta-band were stronger in the feedback direction (Bastos et al., 2011, 2015). For a review of these findings in relation to current models of the function of oscillations in inter-areal neuronal communication, see (Bastos et al., 2014b).

These dissociations in forward and backward connections are particularly important from the theoretical view of predictive coding: in these models of hierarchical inference, the role of forward connections is to transmit prediction error from earlier levels to later levels, whereas the role of backward connections is to transmit predictions from later levels to earlier levels (Friston, 2008; Rao and Ballard, 1999). In predictive coding, backward predictions serve to constrain or explain away activity of earlier levels that is consistent with the internal generative model. In other words, top-down predictions reduce prediction errors that reflect mismatch between those predictions and bottom-up sensory input. Because of this, their polysynaptic effective connectivity is assumed to be suppressive or inhibitory. This proposed inhibitory effect of feedback is supported by many studies of feedback connections (reviewed in Bastos et al., 2012). The predictive coding model also proposes an asymmetry in terms of fast and slow frequencies: backward connections transmitting high-level model predictions must—by their nature—use slower frequencies than the forward connections transmitting prediction errors (Friston, 2008). Heuristically, this spectral asymmetry is inherited from a fundamental property of predictive coding: in predictive coding, predictions (from deep pyramidal cells) accumulate prediction errors (from superficial pyramidal cells). This accumulation effectively performs a gradient descent, such that the rate of change of deep pyramidal cells is driven by ascending prediction error. This means that the frequencies of prediction errors are attenuated in proportion to their frequency in terms of the neuronal responses they elicit (Bastos et al., 2012). Conversely, descending predictions suppress prediction errors directly, thereby preserving their frequency content. This means that high frequencies in ascending prediction errors are suppressed at successive levels of hierarchical processing, in contrast to descending predictions.

This dissociation, with fast frequencies relaying prediction errors and slower frequencies relaying predictions, is consistent with studies that have examined oscillatory responses during repetition suppression, auditory processing of expected and unexpected stimulus sequences, and auditory/visual speech processing (Arnal et al., 2011; Bauer et al., 2014; Kaliukhovich and Vogels, 2012; Todorovic et al., 2011). Recently, Bauer et al. (2014) characterized attention-dependent changes in alpha activity and gamma activity in human subjects using MEG. They found that whereas increases in alpha attentional lateralization tracked stimulus predictability, gamma attentional lateralization was suppressed by stimulus predictability. These results suggest that slower frequencies (alpha) may be used to convey predictions, while faster frequencies (gamma) could reflect prediction errors. For a review of these findings in relation to predictive coding, see Friston et al., 2014b.

To test these predicted asymmetries in message passing between cortical levels, it is useful to consider how neural microcircuits generate oscillations, and how intrinsically coupled microcircuits interact through extrinsic connections. In the next section, we will introduce a dynamic causal model of canonical microcircuits that was designed to address the above issues by specifying how intrinsic and extrinsic connections shape oscillatory coupling. Our hope was that this would allow us to model in greater detail the asymmetries (in terms of anatomical connections, functional valence, frequency content, and putative function) that distinguish forward and backward connections.

### Dynamic causal modeling with canonical microcircuits

In this section, we briefly review the theoretical and empirical evidence that computations throughout the cortex can be understood as canonical. We introduce a DCM that captures many functional and anatomical properties of canonical microcircuits that constitute the cortex and that importantly contains the requisite connections to perform hierarchical Bayesian filtering (predictive coding). Dynamic causal models of this sort allow real data from invasive (e.g., LFP/ECoG) or non-invasive (e.g., EEG/MEG) recordings to be used to characterize the canonical microcircuits that generated them. We will see an example of this in subsequent sections.

It is useful to consider the massive computational problem that any cortical column faces in transforming synaptic inputs, performing computations on those inputs, and relaying the results of these computations as spiking outputs to other cortical or subcortical areas. First, the number of afferent synaptic inputs is typically small compared to the number of intrinsic (local) synaptic input. For example, in cortical area V1, only 4% of all the synapses in the granular (main input) layer are from the LGN—most of the remaining synapses are intrinsic connections from the local column (Binzegger et al., 2004). In addition, when a retrograde tracer is injected into a given cortical area, 70–90% of labeled neurons are contained within the immediately adjacent (within 2 mm) cortical patch, meaning that the bulk of neuronal interactions occur intrinsically, and extrinsic inputs are vastly outnumbered by intrinsic inputs (Markov et al., 2012). The cortex needs mechanisms to effectively select and sustain these sparse inputs in order to have a chance at performing any useful computation on them. Second, as the cortex amplifies these inputs, strict homeostatic circuit properties must be in place to constrain excitation relative to inhibition—to prevent runaway excitation (as observed experimentally by Haider et al., 2006). Third, a given cell in the cortical column must be able to effectively select relevant synaptic inputs from a massive number of potential presynaptic signals, since a given pyramidal cell in the cortex receives about 10,000 synapses (Larkman, 1991). Lastly, in order to functionally segregate top-down from bottom-up processing, a given column must be able to separate higher-order inputs from lower-order inputs—which appears to be accomplished at least in part by the laminar termination pattern of synaptic inputs (Felleman and Van Essen, 1991), and in part by functional segregation as discussed in more detail below. Inputs to a cortical column from cortical areas above it in the hierarchy could, through their larger sampling of the perceptual field and their more elaborated response properties, convey signals that contextualize signals from earlier areas. These computational challenges are faced by nearly all cortical areas—if a solution to these issues arose during evolution, it seems likely that it would be conserved over many species and present, to some extent, in all cortical circuits.

Rodney Douglas and Kevan Martin proposed a “canonical microcircuit for the neocortex” based on their recordings from cat primary visual cortex that contains the necessary properties to meet these computational demands (Douglas et al., 1989; Douglas and Martin, 1991, 2004). Furthermore, they hypothesized that elements of this circuit could be replicated, with minor variations, throughout the cortical sheet. In their model, weak thalamic inputs project onto a cortical column containing three cell populations: excitatory cells in the superficial and deep cortical layers, and a common pool of inhibitory interneurons. Through intrinsic interconnections among these populations, weak thalamic inputs are amplified. Reciprocal connections between the populations maintain a balance of inhibition and excitation. Relatively strong connections between the inhibitory cells and deep pyramidal cells segregate the superficial and deep cell responses. Lastly, in their revised model, dense (lateral) interconnections in the superficial pyramidal cells allow these cells to sample their diverse inputs on the dendritic tree and implement a version of a winner-take-all algorithm (Douglas and Martin, 2004). The results of this computation are transferred to lower cortical areas via the deep pyramidal cells or to higher cortical areas via the superficial cells.

From the theoretical point of view of hierarchical inference in the brain, a canonical microcircuit for predictive coding has also been proposed recently (Bastos et al., 2012). This computational treatment appeals to the free energy principle, which provides a set of mathematical constraints for how homeostatic, biological systems like the brain must operate. In brief, the argument goes as follows (see Friston, 2008 for an in-depth treatment): biological systems must exist within a bounded (physiological) set of states to ensure their survival. To accomplish this, biological systems must minimize the entropy of the probability distribution of their states, over time, which corresponds to minimizing surprise at each point in time. Minimizing surprise is the same as maximizing Bayesian model evidence. Under some simplifying assumptions, this corresponds to minimizing prediction error under a (generative) model of sensory inputs—in other words, the brain tries to predict its sensations using a model with the greatest evidence (minimum complexity). Clearly, in order to accomplish this, the brain must contain a generative model of its environment. Self-evidently, the causes and contexts of the sensory states that our brains sample interact in a hierarchical and nonlinear fashion—a simple example of this is visual occlusion, in which the cause of one percept (the occluded object) interacts with its context (the occluder). In a typical visual scene, multiple causes and contexts at different levels interact with one another and change dynamically to produce sensory input, which poses a complex inference (inverse) problem for the brain. In order to cope with such conditional dependencies at multiple levels and time scales, the brain must possess a hierarchical generative model whose structure is able to represent the contents, causes, and contexts of the world in which it finds itself.

**Fig. 1:**
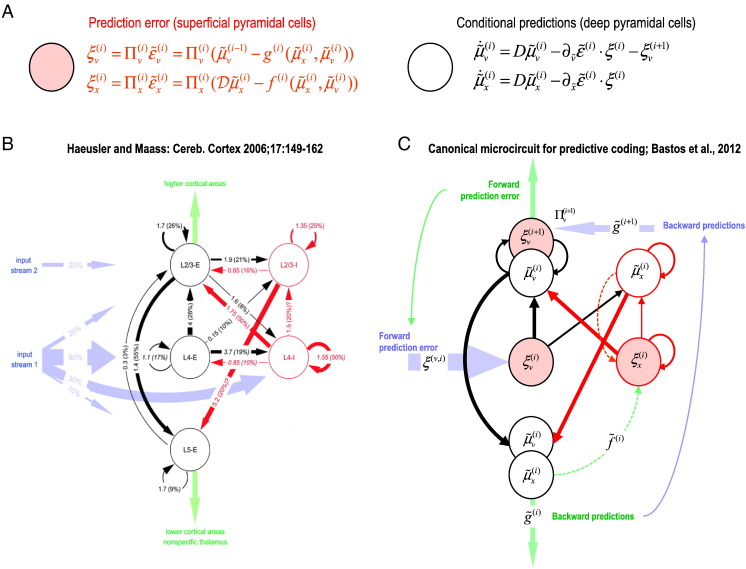
Panel A: The equations in the upper panels define the dependencies between prediction error and conditional estimates (*μ*) of hidden states (*x*) and hidden causes (*v*). These equations have a general form and correspond to generalized predictive coding or Bayesian filtering. The precise form of these equations has been described in many previous communications (see Friston, 2008, for details). In brief, prediction errors are formed at each level of the hierarchy on the basis of conditional estimates at the current level and top-down or lateral predictions based on conditional estimates at the same level or a higher level. These prediction errors are weighted by their estimated precision (inverse variance) and are combined to drive conditional estimates in the same level and the level above. The top-down predictions are formed through nonlinear functions *f* and *g*) of conditional estimates that constitute the hierarchical generative model that is implicit in the connectivity. The relevant point for the present study is the form of these equations and the implicit dependencies among the various terms, which require physical (intrinsic and extrinsic) connections in the brain. Panel B: An overview of intrinsic circuitry based on neuroanatomical and functional data based on a review of quantitative studies by Haeusler and Maass (2007). Panel C: Our interpretation of this canonical connectivity establishing the relationship between the quantities in the predictive coding scheme (top panel) and specific cell populations in canonical microcircuits (Bastos et al., 2012 for details).

Predictive coding is a scheme that embodies these features of generative models. In short, it proposes that the brain minimizes surprise (prediction errors) by inverting a hierarchical generative model to recover Bayesian or conditional estimates of the hidden causes of its sensations (describing the relevant states of its biological eco-niche). These estimates correspond to predictions about hidden states and causes at multiple hierarchical levels. In addition to conditional estimates, predictive coding schemes maintain that the brain also represents prediction errors, which correspond to the difference between the conditional estimates at one level of the hierarchy and those predicted by the level above. At the lowest level, this corresponds to sensory prediction error, namely, the difference between predicted and actual sensations. Fig. 1 provides a schematic describing this sort of predictive coding in terms of differential equations. These equations (Fig. 1A) find the best possible explanation (in a Bayesian sense) for sensory inputs. Bastos et al. (2012) exploits the remarkable correspondence between the computational dependencies implied by this scheme and the intrinsic and extrinsic canonical circuitry (Fig. 1B) to associate specific cell types with specific computational roles. The end point of this exercise is a canonical microcircuit for predictive coding (Fig. 1C).

**Fig. 2:**
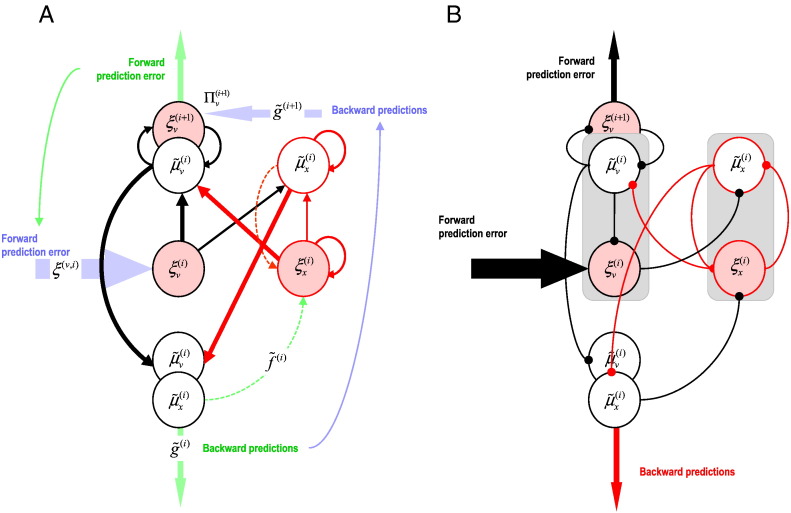
Two pairs of cell populations were combined in moving from the full model (panel A) to the reduced model (panel B). Effectively, we simply absorbed excitatory interneurons in the superficial layers into the excitatory cells of the granular layer, and similarly for inhibitory interneurons.

The arrangement in Fig. 1C can be regarded as a proposal for a canonical microcircuit for predictive coding. However, in this form, the model is slightly over-parameterized for dynamic causal modeling of electromagnetic responses (EEG/MEG/LFP). Usually, dynamic causal models only entertain three subpopulations or neuronal masses (David et al., 2006; Garrido et al., 2007; Moran et al., 2008, 2011). In the current paper, we describe a dynamic causal model with four subpopulations and show that this additional complexity can still be identified using simulations and neurophysiological data. Therefore, we conclude this section by describing a reduced canonical microcircuit with four cell types that will be used to model LFP data in subsequent sections. This canonical microcircuit model has already been used to model EEG and MEG evoked potentials (Brown and Friston, 2012, 2013; Fogelson et al., 2014; Moran et al., 2013) and ECoG within-area cross-spectral densities (Pinotsis et al., 2014). Here, we provide the theoretical and computational motivation for the canonical microcircuit DCM used in these and the current study:

In brief, we set ourselves the constraint that the model should only use four sets of second-order ordinary differential equations (as in the Jansen and Rit model). We therefore chose to model the conditional expectations and prediction error units for hidden causes and states, respectively. This reduction involved collapsing two pairs of cell types in the full model (see Fig. 2A) to arrive at a reduced model (Fig. 2B): the conditional expectation of hidden causes ${\tilde{\mu }}_{v}^{\left(i\right)}$ and the prediction error on hidden causes ${\tilde{\xi }}_{v}^{\left(i\right)}$ are represented by the conditional expectation of hidden causes ${\tilde{\mu }}_{v}^{\left(i\right)}$. Similarly, the conditional expectation of hidden states ${\tilde{\mu }}_{x}^{\left(i\right)}$ and the prediction error on hidden states ${\tilde{\xi }}_{x}^{\left(i\right)}$ are represented by the prediction error of hidden states ${\tilde{\xi }}_{x}^{\left(i\right)}$. Note that the reduction has not eliminated any representations, but has simply removed populations that were represented in two locations in the full model. This choice was somewhat arbitrary but parsimoniously collapses excitatory cells in the granular and supragranular layers into a single population and, similarly, inhibitory cells in the granular and supragranular layers. This preserves the topology of the extrinsic connectivity (superficial cells giving rise to forward connections and deep cells giving rise to backward connections), while producing a simplified model that we know can be identified with reasonable efficiency given typical data.

**Fig. 3:**
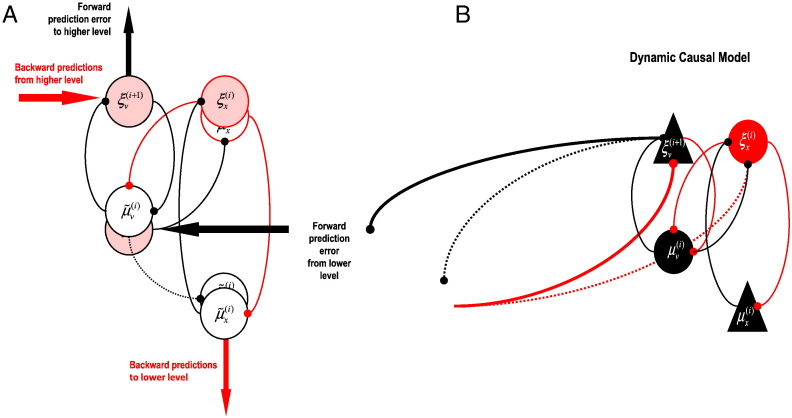
Panel A: The reduced canonical microcircuit of the previous figure, shown in terms of its constituent computational representations. Panel B: The corresponding dynamic causal model, where triangles represent pyramidal cells giving rise to extrinsic connections to other cortical columns and circles represent local interneurons that project only intrinsically. The red circle represents the inhibitory interneuron population, while red lines represent inhibitory connections between populations. Black circles and triangles denote excitatory (either pyramidal or interneuron) populations, and black lines excitatory connections between populations. Note that a few excitatory (in black) populations give rise to inhibitory (in red) connections—we imagine that these are implemented via (unmodeled) inhibitory populations (see text for more details). Not shown in the figure are the self-connections of each population, which are inhibitory.

Note that in the DCM (Fig. 3B), we have changed the excitatory intrinsic connections of the superficial pyramidal cells onto the layer 4 input cells to be inhibitory, and additionally, the backward connections from deep pyramidal cells to the superficial pyramidal and inhibitory interneurons of the previous cortical column are also inhibitory. These flips in the signs of the connections ensure that each pair of interconnected subpopulations has one inhibitory connection and one excitatory connection. This ensures that inhibition and excitation are balanced. Technically, imposing this anti-symmetry on the intrinsic connections ensures that the microcircuit has a fixed-point attractor. This enables us to use a local linear stability analysis at the fixed point to predict spectral responses. A fixed point is important when it comes to predicting spectral responses because these predictions rest upon an expansion around the system's fixed point (see Moran et al., 2008). Clearly, this limits the class of models that can be entertained in explaining data; however, simulations under different parameters suggest that the dynamic repertoire of this class is more than sufficient to reproduce spectra typically seen empirically.

**Fig. 4:**
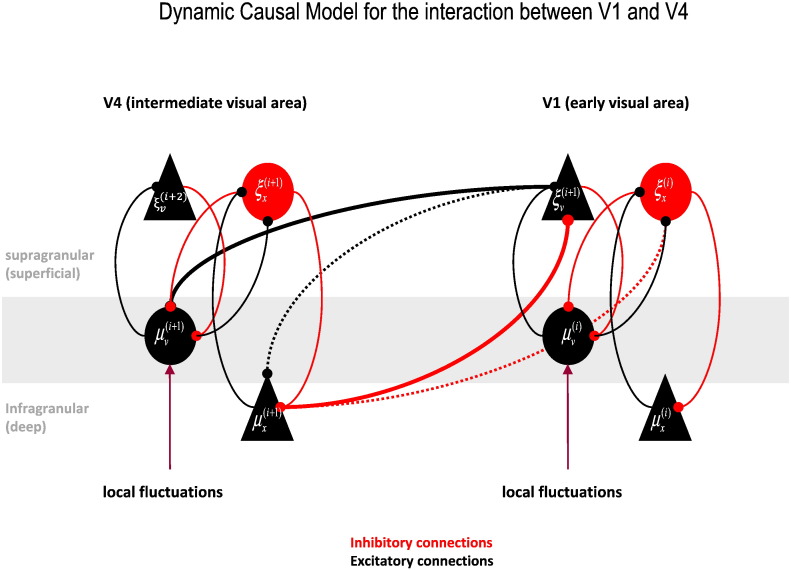
This figure shows the full (intrinsic and extrinsic) circuitry between two areas; here, V1 (a hierarchically earlier area) and V4 (a later area). This model will be used later to model real data. Red connections are inhibitory and black connections are excitatory. Each area receives endogenous drives or fluctuations (arrows) that enter the layer 4 input cells (spiny stellate cells).

Furthermore, an overall (polysynaptic) inhibitory role for backward connections (see Fig. 3B, extrinsic feedback connections) is also consistent with anatomy and physiology (as reviewed in the previous section) and is prescribed by the equations in Fig. 1 (prediction error units on hidden causes ${\tilde{\xi }}_{v}^{\left(i\right)}$ are the weighted *difference* of current causes ${\tilde{\mu }}_{v}^{\left(i-1\right)}$ and higher-order predictions ***g***^(*i*)^). In DCM, inhibitory connections that originate from excitatory cells are imagined to be mediated indirectly via inhibitory interneurons. This applies to the self-inhibition (not shown) of excitatory cells—such as self-inhibition of superficial pyramidal cells. The ensuing reduced model is shown in Fig. 3A in terms of its computational representations and in terms of its constituent cell types in Fig. 3B. This model will be used to model the cross spectra from LFP signals recorded in V1 and V4 in the last section (see Fig. 4). First, we consider how a DCM—of the sort shown in Fig. 4—is identified (inverted) using empirical data.

### Dynamic causal modeling of cross-spectral densities

The goal of DCM is to account for observed neurophysiological data features in terms of the underlying circuits that caused them. In more traditional approaches to data analysis in neuroscience, data features such as event-related potentials (ERPs), induced or evoked power spectra, or coherence spectra between signals are examined and compared in the context of a particular task. In DCM, those same data features are taken as a starting point: one performs Bayesian model inversion using a biophysically plausible generative model, in order to select an optimal model that explains the observed data features. An optimal model is a model that has the greatest Bayesian model evidence; in other words, a model that fits the data accurately but with the minimum of complexity. Minimizing complexity means that the parameters of the model should deviate from prior beliefs as little as possible. Model inversion or fitting provides approximate posterior estimates of model parameters and a free energy approximation to the model evidence. This is generally used to compare competing explanations or models of the same data.

The generative model used by DCM usually comprises two parts: (i) the neuronal model, which comprises equations describing the dynamics of hidden neuronal states and their responses to exogenous or endogenous input, and (ii) an observation model mapping hidden neuronal states (source-level activity) onto observed electrophysiological responses (at the sensor level). For EEG and MEG data, the observation model would specify how source activity is volume conducted to the scalp level (for example, assuming a current-source dipole model), whereas for LFP recordings, the observation model includes the parameterized channel-specific gain and assumptions about the spectral form of (channel-specific and nonspecific) noise associated with the recordings. For the neuronal model, in this paper, we have chosen differential equations based on synaptic convolution, as in the Jansen and Rit model (Jansen and Rit, 1995). In these models, the dynamics of each neuronal mass or subpopulations are modeled with two transformations: the transformation of presynaptic input to postsynaptic depolarization and the transformation of depolarization to spiking output. The latter (postsynaptic potentials to spiking) transformation is approximated using a sigmoid activation function, which approximates a nonlinear transformation of voltage to spike rate, averaged over an ensemble of neurons. The first transformation (spiking to postsynaptic potentials) is approximated by convolving a synaptic alpha kernel (either inhibitory or excitatory) with incoming spikes. Separate neuronal masses are used to model the activity of separate neuronal subpopulations of a cortical column or source. In our previous papers using generative models based on neural masses, we have assumed three subpopulations: these comprise input cells (spiny stellate cells), inhibitory cells, and pyramidal cells, which give rise to extrinsic connections to other cortical areas (e.g., Moran et al., 2008). In the biophysical model used in this paper, we have four cell populations (see Fig. 4), with intrinsic and extrinsic connections that conform to the canonical microcircuit, reviewed in the previous section.

This resulting neuronal model—specified by differential equations—describes the neuronal dynamics. When these equations are linearized around their fixed point, they specify the transfer functions from endogenous input (to the spiny stellate populations) to depolarization in each population. When supplemented with the observation model, the transfer functions map from endogenous fluctuations at each source to observed responses in each channel. Given the form of the spectral density of the endogenous fluctuations, these transfer functions determine the complex cross-spectral density matrix over measured sites. Recently, we have extended the model inversion procedure used by DCM to make use of both the absolute value and argument of the cross-spectral density matrix (Friston et al., 2012). This is important because it allows us to estimate the underlying coherence spectrum (the absolute value of the cross-power spectrum divided by the square root product of the two auto-power spectra), the phase-delay spectrum (argument of the cross-spectral density), and the cross-correlation function (the inverse Fourier transform of the cross-spectral density matrix). Crucially, because these estimates are based on biophysical parameters, they are not contaminated by noise. In other words, these Bayesian estimators of coherence and phase delay are essentially what one might have observed—at the sensor or source level—in the absence of measurement noise. Furthermore, these conditional estimates have a form that can be produced by realistic neuronal circuits. In addition, a large number of different connectivity metrics (beyond coherence) such as phase-locking value, phase-slope index, imaginary coherency, and nonparametric Granger causality can be derived directly from the cross-spectral density matrix (Dhamala et al., 2008; Friston et al., 2014a; Lachaux et al., 1999; Nolte et al., 2004, 2008). In short, DCM for cross-spectral densities enables us to make inferences about the biophysical microcircuits that generate different patterns of observed functional connectivity measured on LFP/MEG/EEG data and then reconstitute the underlying transfer functions (and related measures) using Bayesian estimates of intrinsic and extrinsic connection strengths.

**Fig. 5:**
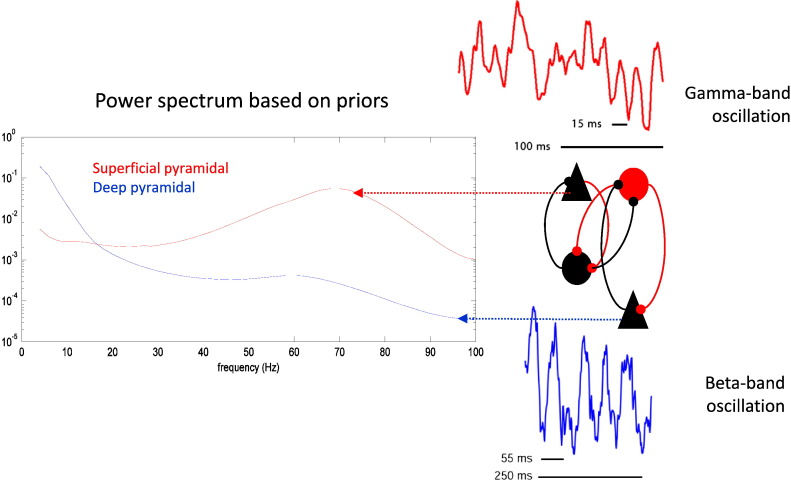
Priors on the model endow superficial pyramidal cells with greater gamma power than the deep pyramidal cells and deep pyramidal cells with more alpha/beta power than superficial pyramidal cells. The auto spectra on the right were evaluated for a single source, using the prior values for coupling parameters and various synaptic rate and time constants (see Tables 1a–1c).

In addition to the intrinsic and extrinsic connectivity structure among the four neuronal masses (per source) that we propose in this paper, an important component of this model rests on the prior distributions over the model parameters, which constrain their dynamics. We have chosen prior values on the intrinsic connections (see Tables 1a–1c) that generate more gamma-band activity in the superficial pyramidal cells relative to the deep pyramidal cells while simultaneously generating more low-frequency (alpha/beta) power in the deep pyramidal cells relative to the superficial cells (Fig. 5). This was motivated by multiple neurophysiological studies that have observed this spectral dissociation between superficial and deep layers of the cortical column (Bollimunta et al., 2011; Buffalo et al., 2011; Maier et al., 2010; Roopun et al., 2006; Smith et al., 2013; Van Kerkoerle et al., 2014; Xing et al., 2012) as well as a dissociation in the auto-correlograms of cells in superficial and deep cortical laminae (Livingstone, 1996). In the final section, we use the described dynamic causal model to characterize forward and backward message passing in spectral terms.

### Empirical analysis

In this section, we use the dynamic causal model of the previous section to analyze data recorded from 15 pairs of sources located in primary visual (V1) and extrastriate (V4) cortical areas of an awake, behaving macaque monkey. Before presenting the results, we first establish that the model had face validity; this means that it can properly identify the correct hierarchical order of two cortical sources using just their observed cross spectra. We then use Bayesian model comparison to confirm that the DCM has predictive validity, in relation to known anatomy: to do this, we compared models of cross spectra between V1 and V4, in which the forward and backward connections were anatomically veridical or reversed. Finally, we use the veridical model parameters to quantify the spectral behavior and transfer functions associated with the forward projecting superficial pyramidal cells and the backward projecting deep pyramidal cells. Our particular interest here was to test whether the frequency content of these populations and their associated transfer functions supports the hypothesis that gamma frequencies transfer information forward and beta frequencies transfer information back.

## Data and experimental setup

All procedures were approved by the ethics committee of the Radboud University, Nijmegen, Netherlands.

#### Experimental paradigm

**Fig. 6:**
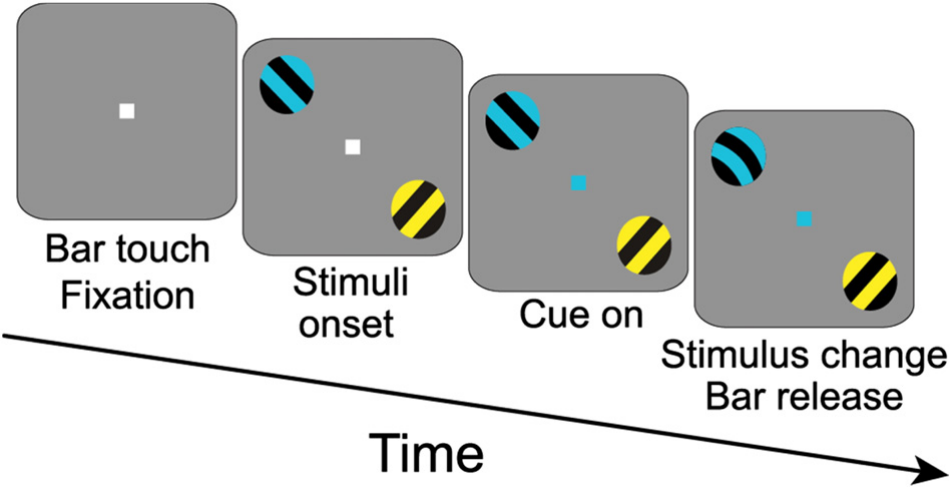
Task design. After touching a bar, the acquisition of fixation, and a pre-stimulus baseline interval of 0.8 s, two isoluminant and isoeccentric stimuli were presented. In each trial, the light grating stripes of one stimulus were slightly tinted yellow, and the stripes of the other stimulus were slightly tinted blue, assigned randomly. After a variable amount of time (0.8–1.3 s), the color of the fixation point changed to blue or yellow, indicating the stimulus with the corresponding color to be the behaviorally relevant (attended) one. We analyzed data averaged across both attention conditions starting 0.3 s after cue onset until the first shape change in one of the stimuli. See Methods for details.

A macaque monkey was trained to perform a visual spatial attention task. After touching a bar, the acquisition of fixation, and a pre-stimulus baseline interval of 0.8 s, two isoluminant and isoeccentric stimuli (drifting sinusoidal gratings, diameter: 3°, spatial frequency: ~ 1 cycles/degree; drift velocity: ~ 1°/s; resulting temporal frequency: ~ 1 cycle/s; contrast: 100%) were presented on a CRT monitor (120 Hz refresh rate non-interlaced). One of the stimuli was presented in the lower right visual hemifield, contralateral to the recorded hemisphere, and the other stimulus was in the upper left hemifield, ipsilateral to the recorded hemisphere. In each trial, the light stripes of one grating were tinted yellow, the light stripes of the other grating tinted blue—assigned randomly and counterbalanced over the spatial locations (right or left). After a variable amount of time (0.8–1.3 s), the color of the fixation point changed to blue or yellow, indicating the grating with the corresponding color to be the behaviorally relevant stimulus. A trial was considered successful (and the monkey rewarded) when it released the bar within 0.15–0.5 s of a change in the cued stimulus, ignoring the non-cued stimulus. The stimulus change comprised a gentle bend in its stripes, lasting 0.15 s. Either one of the two stimuli could change at a random time between stimulus onset, and 4.5 s after cue onset. Trials were terminated without reward, when the monkey released the bar outside the response window, or when it broke fixation (eye position within 1–1.5° radius of the fixation point). For the analyses presented here, data from all correct trials of both attention conditions were pooled. See Fig. 6 for a graphical depiction of the task.

#### Neurophysiological recordings

Neuronal signals were recorded from the left hemisphere in one monkey using subdural ECoG grids consisting of 252 electrodes (1 mm diameter), which were spaced 2–3 mm apart (Rubehn et al., 2009). The grids were implanted under aseptic conditions with isoflurane anesthesia. Intra-operative photographs were acquired for coregistration with the anatomy. Signals were amplified, low-pass filtered at 8 kHz, and digitized at 32 kHz. Local field potentials were obtained by low-pass filtering at 250 Hz and down sampling to 1 kHz.

#### Data analysis general

We computed bipolar differences from neighboring electrodes, to enhance the spatial specificity of the signals, and to remove the common recording reference. We refer to the bipolar differences as “sites.” For the current analyses, we use the time period from 0.3 s after the cue onset (the change in the fixation point color) until the first change in one of the stimuli, from trials with a correct behavioral report. This period constitutes the relatively stationary period of well-defined attentional set. For each trial, this period was cut into non-overlapping 0.5 s data epochs. For each site and recording session, the data epochs were *z*-scored (mean subtracted and divided by their standard deviation), and subsequently, the data epochs were pooled across the recording sessions. This resulted in 1746 data epochs per attention condition. We averaged across both attention conditions in this study. The effects of attention will be reported in a separate communication. Power-line artifacts at 50, 100, and 150 Hz were estimated and subtracted from the data, and epochs containing artifacts were removed with a semi-automatic artifact rejection protocol, based on a variance threshold.

#### Region of interest (ROI) definition

Sites were assigned to different areas based on their positions as seen in the surgical pictures. We used the areal boundaries according to Saleem and Logothetis (2007). This yielded 63 sites in V1, 16 in V4. For the analyses of this paper, we use 15 V1–V4 site pairs that showed strong gamma-band coherence. This selection step ensured that the site pairs we studied received strong bottom-up drive, given the receptive field locations of the neurons underlying the site pairs and the position of the visual grating stimulus (Bosman et al., 2012). Note that the selection of particular channels of invasive data is not an inherent aspect of applying DCM. Although we are using carefully selected invasive data to demonstrate this particular DCM, it can also be used to model non-invasive (human) data, where the electrode gains are replaced by a conventional electromagnetic forward model.

## Face validation using simulated data

Face validation just means that model optimization and selection does what it is supposed to. In this context, we need to establish that the correct direction of forward and backward connections can be inferred from empirical cross spectra. Clearly, this can only be established when one knows the true direction of the connections; in other words, by using simulated data produced by known forward and backward connectivity. However, these simulated data should be generated using realistic connectivity parameters that are representative of real neuronal circuits. We ensured this by simulating cross spectra using the parameter estimates obtained by fitting the model to real data (these are the parameters reported in the empirical results section later). These spectral data were then mixed with (complex) noise with a log precision of seven: in other words, the ratio of simulated signal variance to noise variance was exp(7). Log precision is therefore equivalent to measuring signal-to-noise in decibels (to within a scaling factor). The simulated data used parameter estimates from anatomically veridical models, giving 15 simulated data sets. We then fitted a model with veridical connections and reverse connections to these simulated data, to see if model comparison could identify the correct model.

**Fig. 7:**
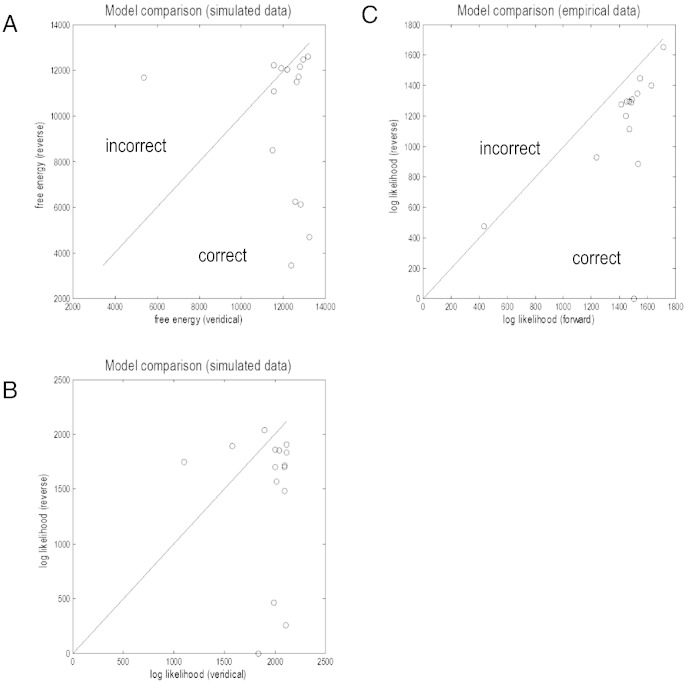
Panels A and B: Results of face validity test. The dots correspond to 15 different V1–V4 channel pairs. Cross spectra were simulated using realistic parameters, and inverted using either the correct hierarchical connections or the reversed connections. Resulting free energies (panel A) and accuracies (panel B) are shown for the two models by plotting them against each other. Ideally, we would expect all the dots to fall beneath the diagonal line. We present the differences in free energy in this slightly unusual way to illustrate how they depend upon the absolute values. Usually, when presenting the results of Bayesian model comparison, one would simply show bar charts of relative free energy. These free energy differences are the vertical distance of any point from the identity line. In the current format, one can see that incorrect differences are limited to data sets with a smaller free energy or log evidence. One should not over interpret this because the evidence for different models should always pertain to the same data. However, given that the data sets, we used were of the same cardinality, the variation in log evidence may reflect something about data quality. Finally, note that the log likelihood is a measure of accuracy (panel B). This means that the differences between panels A and B can be attributed to complexity. Panel C: Results of predictive validity test, using the same format as the previous panels except the results use real data and test whether the model has more evidence for the correct compared to incorrect pattern of connectivity. The dots correspond to 15 different V1–V4 channel pairs. Cross spectra were fitted with either the correct connectivity (forward projections from V1 to V4, backward projections from V4 to V1) or reversed connectivity (forward projections from V4 to V1, backward projections from V1 to V4), and the resulting model accuracies are shown.

Our provisional model comparison used the free energy approximation to the log evidence as described in the previous section. The results of this model comparison are shown in Fig. 7A by plotting the free energy of the reverse model against the veridical model. If model selection is working properly, the resulting points should lie to the right of the identity line. Displaying the results in this form allows one to see whether there is any systematic difference in model comparison between inversions that have a high and low free energy (log evidence). It can be seen from Fig. 7A that the performance of free energy was qualitatively reasonable (12 out of 15 correct), but quantitatively disappointing, in that the difference in free energy exceeded several thousand for a data set that was incorrectly identified (a free energy difference of three would normally be taken as strong evidence in favor of the winning model). This large free energy difference may be attributable to precise priors on the precision (signal-to-noise ratio) that can cause the complexity part of the log evidence to dominate over the accuracy part (expected log likelihood). One can finesse this problem by examining the accuracy, under the assumption that the complexities of the two models are equivalent. The resulting approximation is motivated easily, given that both models used exactly the same number of parameters (and priors on those parameters). Furthermore, we can partition the accuracy into auto-spectral and cross-spectral components (i.e., the log likelihood of the auto-spectral and cross-spectral data features) and focus on the cross spectra, which contain the information that informs the estimates of extrinsic connections between sources. In summary, this leads to the following approximation for the log evidence

$$\begin{array}{c} F_{ij}=E_{Q}\left[\ln p\left(y_{ij}|\vartheta ,m\right)\right]\\ =-\frac{1}{2}{\varepsilon_{ij}}^{T}\Pi \left(\mu \right)\varepsilon_{ij}+\frac{1}{2}\ln|\Pi \left(\mu \right)|\\ \varepsilon_{ij}=\left(y_{ij}-g_{ij}\left(\mu \right)\right) \end{array}$$

where *g*(*μ*)_*ij*_ is the predicted cross-spectral data *y_ij_* between sites *i* and *j*, given the posterior density over model parameters *Q*(*ϑ*) = *N*(*μ*, *Σ*) and *Π*(*μ*) is the noise precision. This approximate log evidence can be pooled and compared in the usual way, where a difference in log evidence of three is considered strong evidence for one model over another; this corresponds to a log odds or likelihood ratio of about 20 to 1 (cf. the *p* = 0.05 criterion in classical inference). Fig. 7B shows this approximation and suggests it is better behaved. Crucially, the three failures of the (15) model comparisons were observed in the models with the lowest log evidence. Although a failure rate of 20% may not seem very impressive, in practice, a model is selected after pooling (adding) the log evidence over all (15) data sets. In this instance, one would have overwhelming evidence for the correct model. The reduced free energy above was used in subsequent model comparisons.

## Predictive validity in relation to anatomy

We then repeated the model comparison above using the 15 pairs of empirical recordings. Fig. 7C shows the results of model comparison, assuming that the correct pattern of connectivity is forward connections from V1 to V4 and backward connections from V4 to V1. Pleasingly, with only one exception, the model comparison identified the anatomically veridical pattern of connections. It can be seen that in the one data set that led to the wrong model being selected, the evidence for this invalid model is trivial, in relation to the valid model. Furthermore, this particular data set was an outlier in the sense that it had the smallest log evidence. Having established the face and predictive validity of the model, we now turn to the quantitative analysis of the parameters of the best (veridical) model:

## Empirical results

**Fig. 8:**
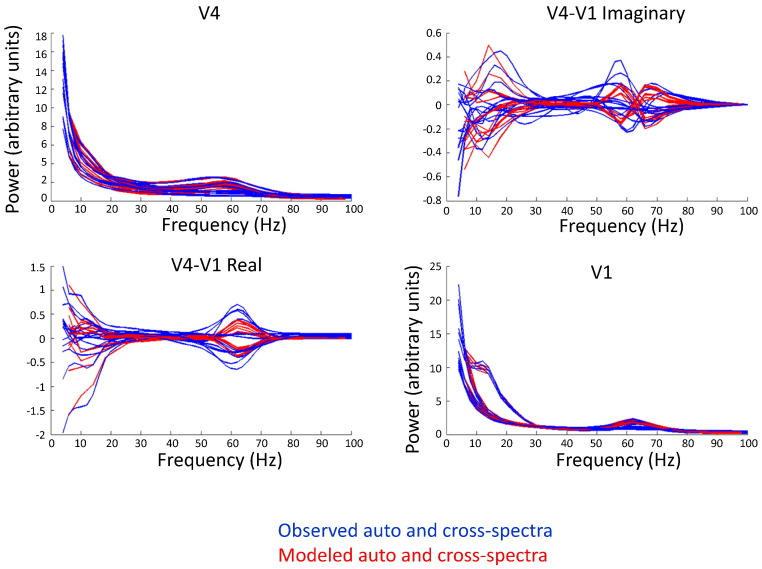
Auto and cross spectra derived from model fits (in red) and neurophysiological data (in blue) for all 15 V1–V4 channel pairs. The V1 and V4 power fits are plotted on the diagonal, whereas the off-diagonal plots represent the cross terms of the cross spectral density matrix—the real part is shown in the bottom left corner, and the imaginary part is shown on the upper right corner.

We first examined the modeled cross spectra for the two sources. Fig. 8 shows the V1 and V4 auto- and cross-spectral densities of the model response (in red), overlaid on the cross spectra of the observed data (in blue). On the diagonal, we have plotted the power estimates (auto spectra), where it is evident that both in V1 and in V4, there is a prominent gamma-band peak between 50 and 70 Hz, a frequency range that is fully consistent with previous studies in visual cortex using similar visual stimuli (Bosman et al., 2012; Fries et al., 2001; Gregoriou et al., 2012). Power also peaks slightly in a subset of V1 sites in the alpha/beta range, and there is also additional energy in this band in V4. Finally, in the off-diagonal terms, the real and imaginary parts of the complex cross spectra—reflecting the inter-areal synchronization between the V1 and V4 sources—also show prominent features around the gamma and alpha/beta frequency ranges. Evidently, the model fits are quite good, and crucially, capture dynamics at both the gamma and alpha/beta frequency ranges.

**Fig. 9:**
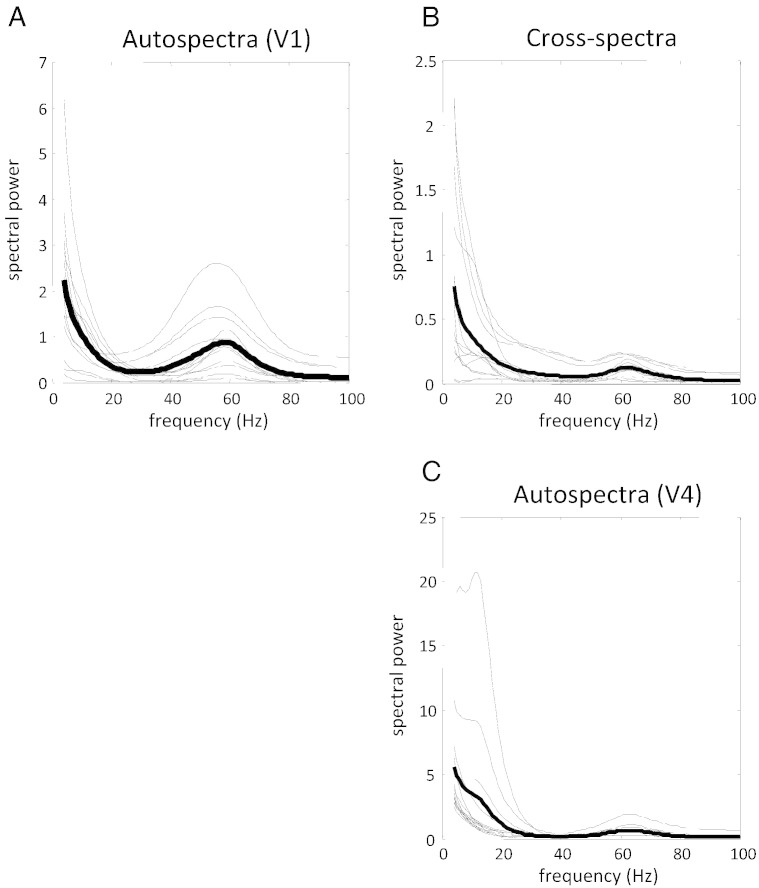
Using conditional parameter estimates from the correct models, here we plot the source-specific power spectra of V1 (panel A), V4 (panel C), and the absolute value of their cross spectra (panel B) after removing the (modeled) effects of channel-specific and -unspecific noise.

These modeled auto and cross spectra reflect the full model output, which is a mixture of both the underlying neuronal dynamics as well as contributions from channel-specific noise (noise that is particular to each recording site and independent across recording sites) and channel-unspecific noise (noise that is common to all recording sites). After fitting the model to the data, we have posterior estimates of how the observed auto and cross spectra are generated and therefore the exact underlying mixture of neuronal dynamics and noise that contribute to the observed LFP. This enables one to remove the estimated noise component from the model's output and see what would have been observed in the absence of noise. In Fig. 9, we show the V1 and V4 auto and cross spectra after removing the effects of channel (specific and unspecific) noise and setting the gain of both (virtual) electrodes to unity. The auto- and cross-spectral densities for all 15 pairs are plotted as dotted lines and the average over pairs are plotted as full lines. It is immediately evident that V1, the source of forward connections to V4, has a pronounced peak in the gamma range (60 Hz). In contrast, the source of backward connections (V4) has a relatively larger peak in the beta range (16 Hz). The (absolute) cross spectra reflect this, with both beta and gamma peaks. See Fig. 8 for the real and imaginary contributions to the absolute values.

**Fig. 10:**
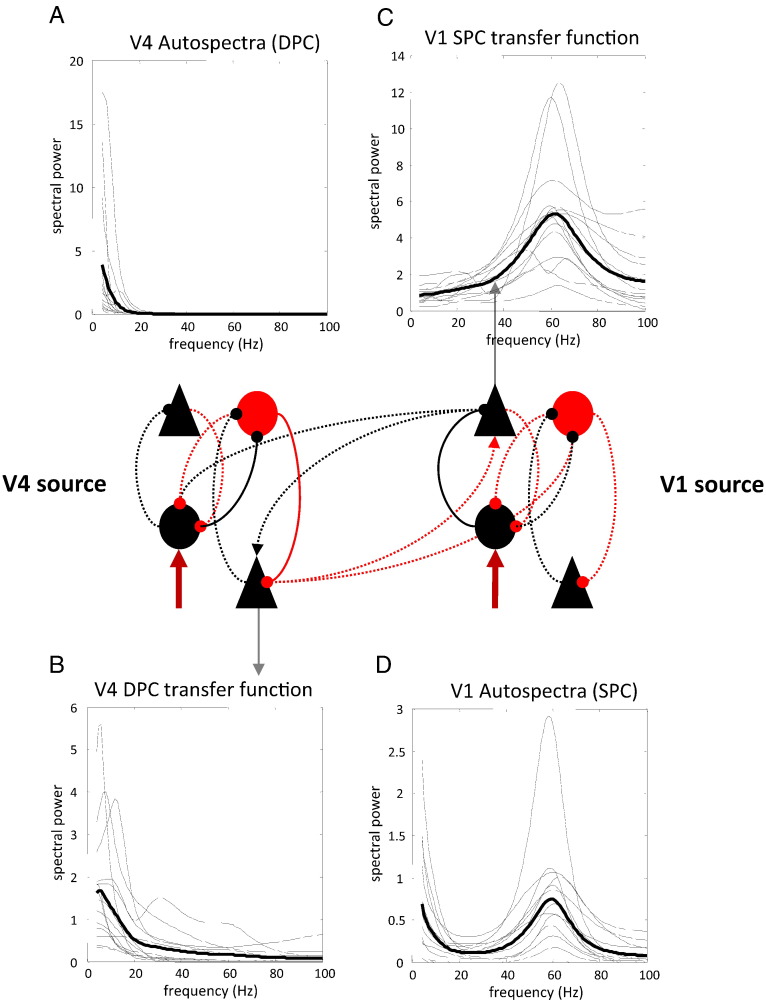
Using conditional parameter estimates from the correct models, this figure shows the source-specific power spectra of the superficial cells of V1 (Panel D) and the deep pyramidal cells of V4 (Panel A). The corresponding transfer functions are shown in the panel C and panel B. These correspond to the transfer functions between local input and superficial pyramidal cells in the lower area (V1) and deep pyramidal cells in the higher area (V4). The intrinsic connections mediating the transfer of power from local fluctuations to the pyramidal cells elaborating forward and backward connections are shown as solid lines (and other connections as dotted lines).

These source-specific reconstructions of spectral activity speak to an asymmetry but they do not tell us precisely which frequencies are communicated from one source to another. This is because the V1 and V4 auto and cross spectra from Figs. 8 and 9 reflect the neuronal dynamics of four interconnected subpopulations. To investigate directed frequency coupling, we need to examine the spectral behavior of the neuronal populations giving rise to forward and backward connections. These populations are the superficial pyramidal cells (SPC) of the V1 source and the deep pyramidal cells (DPC) of the V4 source. We can selectively interrogate the cross spectra between specific subpopulations because DCM fully parameterizes the observation model, which specifies how much each subpopulation contributes to the observed LFP. A priori, we have assumed that the superficial pyramidal cells contribute most of the observed signal (see Table 1c, Watanabe et al., 2012). However, because the observation model is parameterized, it is also optimized during model inversion. Therefore, we can recover the auto and cross spectra that are generated by individual subpopulations by evaluating the cross spectra using an observation model that samples selectively from subpopulations of interest. These features of DCM allow us to investigate the auto- and cross spectra of specific neuronal subpopulations within the modeled V1 and V4 sources. Fig. 10 shows the autospectra of the V1 superficial pyramidal cells and the autospectra of the V4 deep pyramidal cells. Here, we see a pronounced distinction between the population-specific activities, relative to the source-specific activity in the previous figure. The activity in the source of backward connections (DPC in V4, Fig. 10A) is dominated by low-frequency components, while the source of forward connections (SPC in V1, Fig. 10D) has a distribution of frequencies that retains a gamma peak.

However, the spectral profiles above conflate power in frequencies that are specific to each cortical source and power that is circulated between sources by reciprocal connections. For example, the spectral power of the deep V4 cells represents both power that is transferred back to V1 as well as power that is transferred to other subpopulations in V4 through intrinsic connections (namely, the deep cells themselves through self-connections and the inhibitory interneurons). To get a clearer picture of the frequencies that are passed forward and backward, one can examine the transfer function mapping between (source-specific) neuronal fluctuations and the origin of forward and backward connections. These transfer functions characterize the frequencies in the endogenous input (that are unique to each source) that are transferred to the superficial (resp. deep) pyramidal cells and subsequently forward (resp. backward).

These transfer functions are shown in Fig. 10 and reveal that gamma frequencies in V1 are passed to V4, and that lower (alpha/beta) frequencies in the V4 cortical source are sent backwards. Interestingly, some transfer functions from deep V4 pyramidal cells to V1 show peaks in the alpha-band; however, when these peaks are pronounced, they are generally in the beta-band. This decomposition, using transfer functions, suggests that the low frequencies in the superficial pyramidal cells of V1 (lower right panel) are due to reciprocal (low-pass) message passing because they are not present in the transfer functions quantifying the frequencies that originate in V1 (upper right panel). In short, these quantitative results confirm the hypothesis that forward projections utilize gamma frequencies, while backward projections employ alpha/beta frequencies.

## Conclusion

In summary, we have used the new canonical microcircuit dynamic causal model to isolate the frequency-specific contributions to asymmetric and directed coupling between hierarchically deployed sources in the cortex. We first established the significance of these biophysically informed models using Bayesian model comparison and were then able to quantify distributed interactions using transfer functions of the optimized model parameters. The results are consistent with the emerging notion that forward connections send signals in higher frequencies, while backward connections use lower frequencies.

The DCM described in this paper is for time-invariant cross spectral densities. Indeed, DCM for cross-spectral responses is generally portrayed as a model under local stationarity assumptions. However, strictly speaking, these models do not assume stationarity. Although the model generates predictions that would be seen under stationarity assumptions, exactly the same predictions would be obtained with endogenous fluctuations whose spectral density averaged over time corresponded to the spectral density estimated following model inversion. In other words, one can treat the estimated spectral density of endogenous fluctuations—and the cross spectra of the data—as averages over time, under the assumption that the effective connectivity remains constant. In future work, we will relax this assumption and use the current DCM as the basis of a model for induced (and evoked) responses with time varying (state-dependent) connectivity, e.g. due to short-term plasticity and NMDA-dependent changes in synaptic efficacy.

One may ask whether our results are biased by our selection of priors, in which we have a priori specified intrinsic connectivity parameters that generate stronger gamma power in superficial cells compared to deep pyramidal cells, and the opposite for lower frequencies. This choice of priors reflects a well-documented dissociation in the frequency content of superficial and deep cortical laminae. Therefore, including this dissociation in our model exploits the Bayesian aspect of DCM, whereby accumulated evidence can be incorporated as priors in a new model. Nevertheless, one might ask whether this choice of prior values (Tables 1a–1c) has predisposed the spectral asymmetry we have observed *a posteriori*. The answer is no because if the frequency dissociation were not present in the data, our test of predictive validity in relation to anatomical hierarchical relationships would have failed. The fact that overall, we have more evidence for the model with correct arrangement of forward and backward connections (forward from V1 to V4 and backward from V4 to V1) in relation to the reversed model means that this asymmetry was present in our data. In other words, in the absence of a true functional asymmetry (or opposite asymmetry), model selection would not discriminate between the correct and inverted arrangement (or select the wrong model).

In our previous analyses of these data (Bastos et al., 2015), we have used Granger causality to dissociate the frequency-specific correlates of directed coupling. One might ask: What is the advantage of using dynamic causal modeling over Granger causality? The advantages are both technical and conceptual. The technical advantage of DCM is that it does not assume that neuronal fluctuations or innovations are spectrally white. This assumption is made in current formulations of Granger causality and places implausible constraints on the frequency profile of neuronal fluctuations that drive observed activity (Ding et al., 2006). This assumption is inaccurate because innovations or inputs to sources are the outputs of other (unmodeled) sources, whose spectral profile (or autocorrelation structure) has to be self-consistent with the outputs of the modeled sources. DCM resolves this problem by allowing for innovations with colored (parameterized) components that are optimized during model inversion. Additionally, Granger causality is susceptible to signal-to-noise ratio differences between recording channels (Friston et al., 2014a; Nalatore et al., 2007). This is because in the presence of either volume conduction or common drive from a third unobserved area to two sensors, a sufficient difference in signal-to-noise ratio (SNR) in one channel, compared to another, can change the predictive power of one signal in relation to another, resulting in a greater (spurious) Granger-causal influence from the time series with better SNR. In most formulations of Granger causality (but see Nalatore et al., 2007), neuronal innovations and channel-specific or -unspecific noise are not modeled separately. Recently, a method has been proposed, termed the instantaneous feedback strength (IFS), to quantify situations in which a significant amount of shared zero-lag noise (e.g., volume-conducted noise) is present in any two given recording channels (Vinck et al., 2015). In situations where the IFS is strong, it was shown that Granger causality analysis generates an unacceptable level of false positives, and under these conditions, Granger causality should not be applied to data (Vinck et al., 2015). DCM finesses this problem by separately parameterizing and estimating noise contributions that arise from correlated and uncorrelated sources during model inversion. By estimating the observation model and the neuronal model separately, this then allows us to extract features related to between-source connectivity that are unaffected by noise. In principle, this makes it possible to measure Granger causality (and other spectral factors; see, for example, Fig. 10 in the current manuscript) between sources after removing the effects of (modeled) measurement noise (Friston et al., 2014a).

Beyond these technical considerations, the conceptual advantage rests on the fact that Granger causality provides a surface description of (lagged) statistical dependencies among observed time series. In contrast, DCM models the hidden states that cause these dependencies, lending the parameters a biophysical meaning. This means one can isolate specific populations or sources of observed variance and specifically examine their properties in terms of intrinsic power and transferred power. In this paper, we have used this to look at superficial and deep pyramidal cells that send forward and backward projections. In contrast, Granger causality cannot access the hidden sources that generated observed time series because it describes dependencies among sensors and not neural sources. However, one advantage of Granger causality is that it is a data-driven method and therefore requires no prior assumptions or knowledge of the observed system. Therefore, a Granger-causal analysis can be used to inform and guide model-based approaches like DCM, especially in systems where less is known about the underlying circuitry or where data may not be sufficient to constrain a physiologically-based DCM (cf. Litvak et al., 2012). These distinctions mean that functional (Granger) and effective (DCM) connectivity measures will play a complementary role in analyzing dynamic brain networks (Friston et al., 2013). In future studies, we will explore the construct validity (that is, whether similar conclusions can be made with different methods) between Granger causality and DCM and examine to what extent the methods converge as different model assumptions are violated (such as the spectral composition of innovations or differences in SNR) (Friston et al., 2014a).

In this paper, we have proposed a mapping between the anatomically and the physiologically characterized intrinsic and extrinsic connectivity of canonical microcircuits and the connectivity predicted by the free energy principle. This involved mapping computational units thought to encode conditional estimates of hidden states, hidden causes, and their prediction errors onto neurobiological populations in specific cortical layers. The resulting microcircuit contains four subpopulations per cortical column, with the key feature that it includes distinct populations for superficial and deep pyramidal cells, which give rise to forward and backward connections, respectively. Clearly, different mappings between predictive coding and the physiological/anatomical data on canonical microcircuits are conceivable and, in principle, DCM could be used to evaluate these alternative proposals through Bayesian model comparison. As more data on intrinsic and extrinsic circuitry is gathered, and as the physiological characteristics of canonical microcircuits become increasingly elucidated, this knowledge could be incorporated into future DCMs.

The canonical microcircuit DCM we have introduced in this paper enables one to test specific predictions made by the free energy principle and predictive coding about cortical processing in normal and diseased states. This is because we have specified the neuronal infrastructure (canonical microcircuit) that prosecutes the necessary computations, and thereby we can estimate these microcircuit properties by inverting real data recorded under relevant experimental conditions. For example, Brown and Friston recently applied this model to study ERP responses to stimuli of varying contrast and found that—across different contrast levels—the gain of the superficial pyramidal cells (defined as the strength of the parameter controlling self-inhibition of the superficial pyramidal cells) showed predicted contrast-dependent changes (Brown and Friston, 2012). Also, Pinotsis et al. (2014) have extended the canonical mass model to a neural field model and study receptive field properties during a visual attention task and visual stimulation with varying contrasts. In predictive coding models, the gain on the pyramidal cells is associated with the precision of prediction errors, in other words, a representation of uncertainty. Therefore, DCM provides a way for testing hypotheses based on predictive coding during normal and impaired inference (in different patient groups), particularly when any supposed pathophysiology involves the neuromodulatory (or synchronous) postsynaptic gain of superficial pyramidal cells (Moran et al., 2013).

We also hope that the model described in this paper may allow more mechanistic questions about the role of oscillations in cortical processing to be addressed. For example, a prominent hypothesis about alpha oscillations suggests that alpha oscillations suppress task-irrelevant representations (Haegens et al., 2011; Jensen and Mazaheri, 2010). One prediction that naturally flows from this hypothesis is that large-amplitude alpha oscillations might be correlated with enhanced inhibitory processing—one can now test whether alpha amplitude positively correlates with extrinsic or intrinsic effective connectivity or the activity of inhibitory populations within a DCM and examine if the sign of correlation is consistent with an inhibitory role. In contrast, gamma frequency oscillations are thought to facilitate cortical communication, and thereby might be positively correlated with effective connectivity within or between areas (Fries, 2005). Indeed, recently it has been shown that selective attention enhances gamma-band coherence between V1 and V4 (Bosman et al., 2012; Grothe et al., 2012). In principle, DCM could provide a more mechanistic understanding of this phenomenon, by asking which specific circuit properties can explain this increase in coherence, and how this might be related to the enhanced neuronal communication that is thought to be reflected in inter-areal gamma-band coherence.

## Software note

The analyses presented in this paper use algorithms that are available as part of the SPM academic freeware.

## Figures and Tables

**Table 1a:** Priors on intrinsic connectivity values (arbitrary units).

Name of parameter	G1	G2	G3	G4	G5	G6	G7	G8	G9	G10
Corresponding connection	SS➔SS	SPC➔SS	II➔SS	II➔II	SS➔II	DPC➔II	SPC➔SPC	SS➔SPC	II➔DPC	DPC➔DPC
Value	4	4	4	4	4	2	4	4	2	1

Abbreviations: SS: spiny stellate; SPC: superficial pyramidal cell; DPC: deep pyramidal cell; II: inhibitory interneuron.

**Table 1b:** Priors on time constants (in milliseconds).

Name of parameter	T1	T2	T3	T4
Corresponding cell population	Spiny stellates	Superficial pyramidal cells	Inhibitory interneurons	Deep pyramidal cells
Value	2	2	16	28

**Table 1c:** Priors on observation model (contribution of each population to the observed LFP signal). These priors on the observation model essentially assume that excitatory cells in all cells contribute to the observed LFP signal recorded by the ECoG channel, but that excitatory cells in the superficial layers contribute with a larger weight, as recently demonstrated (Watanabe et al., 2012).

Name of parameter	J1	J3	J5	J7
Corresponding cell population	Spiny stellates	Superficial pyramidal cells	Inhibitory interneurons	Deep pyramidal cells
Value	0.2	0.8	0	0.2

## References

[bb0005] Alink A., Schwiedrzik C.M., Kohler A., Singer W., Muckli L. (2010). Stimulus predictability reduces responses in primary visual cortex. J. Neurosci..

[bb0010] Arnal L.H., Wyart V., Giraud A.-L. (2011). Transitions in neural oscillations reflect prediction errors generated in audiovisual speech. Nat. Neurosci..

[bb0015] Barone P., Batardiere A., Knoblauch K., Kennedy H. (2000). Laminar distribution of neurons in extrastriate areas projecting to visual areas V1 and V4 correlates with the hierarchical rank and indicates the operation of a distance rule. J. Neurosci..

[bb0020] Bastos A., Bosman C., Schoffelen J.M., Oostenveld R., Fries (2011). Interareal directed interactions and their modulation by selective attention assessed with high density electrocorticography in monkey. Front. Hum. Neurosci..

[bb0030] Bastos A.M., Usrey W.M., Adams R.A., Mangun G.R., Fries P., Friston K.J. (2012). Canonical microcircuits for predictive coding. Neuron.

[bb0025] Bastos A.M., Briggs F., Alitto H.J., Mangun G.R., Usrey W.M. (2014). Simultaneous recordings from the primary visual cortex and lateral geniculate nucleus reveal rhythmic interactions and a cortical source for γ-band oscillations. J. Neurosci..

[bb0040] Bastos A.M., Vezoli J., Fries P. (2014). Communication through coherence with inter-areal delays. Curr. Opin. Neurobiol..

[bb0035] Bastos A.M., Vezoli J., Bosman C.A., Schoffelen J.-M., Oostenveld R., Dowdall J.R., Weerd P.D., Kennedy H., Fries P. (2015). Visual areas exert feedforward and feedback influences through distinct frequency channels. Neuron.

[bb0045] Bauer M., Stenner M.-P., Friston K.J., Dolan R.J. (2014). Attentional modulation of alpha/beta and gamma oscillations reflect functionally distinct processes. J. Neurosci..

[bb0050] Binzegger T., Douglas R.J., Martin K.A. (2004). A quantitative map of the circuit of cat primary visual cortex. J. Neurosci..

[bb0055] Bollimunta A., Mo J., Schroeder C.E., Ding M. (2011). Neuronal mechanisms and attentional modulation of corticothalamic alpha oscillations. J. Neurosci..

[bb0060] Bosman C.A., Schoffelen J.-M., Brunet N., Oostenveld R., Bastos A.M., Womelsdorf T., Rubehn B., Stieglitz T., De Weerd P., Fries P. (2012). Attentional stimulus selection through selective synchronization between monkey visual areas. Neuron.

[bb0065] Brown H.R., Friston K.J. (2012). Dynamic causal modelling of precision and synaptic gain in visual perception—an EEG study. NeuroImage.

[bb0070] Brown H.R., Friston K.J. (2013). The functional anatomy of attention: a DCM study. Front. Hum. Neurosci..

[bb0075] Buffalo E.A., Fries P., Landman R., Buschman T.J., Desimone R. (2011). Laminar differences in gamma and alpha coherence in the ventral stream. Proc. Natl. Acad. Sci..

[bb0080] Bullier J., Hupé J.M., James A., Girard P. (1996). Functional interactions between areas V1 and V2 in the monkey. J. Physiol. Paris.

[bb0085] Buschman T.J., Miller E.K. (2007). Top-down versus bottom-Up control of attention in the prefrontal and posterior parietal cortices. Science.

[bb0090] Chen C.C., Henson R.N., Stephan K.E., Kilner J.M., Friston K.J. (2009). Forward and backward connections in the brain: a DCM study of functional asymmetries. NeuroImage.

[bb0095] Covic E.N., Sherman S.M. (2011). Synaptic properties of connections between the primary and secondary auditory cortices in mice. Cereb. Cortex.

[bb0100] David O., Kiebel S.J., Harrison L.M., Mattout J., Kilner J.M., Friston K.J. (2006). Dynamic causal modeling of evoked responses in EEG and MEG. NeuroImage.

[bb0105] De Pasquale R., Sherman S.M. (2011). Synaptic properties of corticocortical connections between the primary and secondary visual cortical areas in the mouse. J. Neurosci..

[bb0110] Dhamala M., Rangarajan G., Ding M. (2008). Estimating granger causality from Fourier and wavelet transforms of time series data. Phys. Rev. Lett..

[bb0115] Ding M., Chen Y., Bressler S.L. (2006). Granger causality: basic theory and application to neuroscience. Handb. Time Ser. Anal..

[bb0120] Douglas R.J., Martin K. (1991). A functional microcircuit for cat visual cortex. J. Physiol..

[bb0125] Douglas R.J., Martin K.A.C. (2004). Neuronal circuits of the neocortex. Annu. Rev. Neurosci..

[bb0130] Douglas R.J., Martin K.A., Whitteridge D. (1989). A canonical microcircuit for neocortex. Neural Comput..

[bb0135] Felleman D.J., Van Essen D.C. (1991). Distributed hierarchical processing in the primate cerebral cortex. Cereb. Cortex.

[bb0140] Fogelson N., Litvak V., Peled A., Fernandez-Del-Olmo M., Friston K. (2014). The functional anatomy of schizophrenia: a dynamic causal modeling study of predictive coding. Schizophr. Res..

[bb0145] Fontolan L., Morillon B., Liegeois-Chauvel C., Giraud A.-L. (2014). The contribution of frequency-specific activity to hierarchical information processing in the human auditory cortex. Nat. Commun..

[bb0150] Fries P. (2005). A mechanism for cognitive dynamics: neuronal communication through neuronal coherence. Trends Cogn. Sci..

[bb0155] Fries P., Reynolds J.H., Rorie A.E., Desimone R. (2001). Modulation of oscillatory neuronal synchronization by selective visual attention. Science.

[bb0160] Friston K. (2008). Hierarchical models in the brain. PLoS Comput. Biol..

[bb0180] Friston K.J., Ungerleider L.G., Jezzard P., Turner R. (1995). Characterizing modulatory interactions between areas V1 and V2 in human cortex: a new treatment of functional MRI data. Hum. Brain Mapp..

[bb0165] Friston K.J., Bastos A., Litvak V., Stephan K.E., Fries P., Moran R.J. (2012). DCM for complex-valued data: cross-spectra, coherence and phase-delays. NeuroImage.

[bb0185] Friston K., Moran R., Seth A.K. (2013). Analysing connectivity with Granger causality and dynamic causal modelling. Curr. Opin. Neurobiol..

[bb0170] Friston K.J., Bastos A.M., Oswal A., van Wijk B., Richter C., Litvak V. (2014). Granger causality revisited. NeuroImage.

[bb0175] Friston K.J., Bastos A.M., Pinotsis D., Litvak V. (2014). LFP and oscillations—what do they tell us?. Curr. Opin. Neurobiol..

[bb0190] Garrido M.I., Kilner J.M., Kiebel S.J., Friston K.J. (2007). Evoked brain responses are generated by feedback loops. Proc. Natl. Acad. Sci. U. S. A..

[bb0195] Garrido M.I., Kilner J.M., Stephan K.E., Friston K.J. (2009). The mismatch negativity: a review of underlying mechanisms. Clin. Neurophysiol..

[bb0200] Girard P., Bullier J. (1989). Visual activity in area V2 during reversible inactivation of area 17 in the macaque monkey. J. Neurophysiol..

[bb0205] Girard P., Salin P.A., Bullier J. (1991). Visual activity in areas V3a and V3 during reversible inactivation of area V1 in the macaque monkey. J. Neurophysiol..

[bb0210] Girard P., Salin P.A., Bullier J. (1992). Response selectivity of neurons in area MT of the macaque monkey during reversible inactivation of area V1. J. Neurophysiol..

[bb0215] Gregoriou G.G., Gotts S.J., Desimone R. (2012). Cell-type-specific synchronization of neural activity in FEF with V4 during attention. Neuron.

[bb0220] Grothe I., Neitzel S.D., Mandon S., Kreiter A.K. (2012). Switching neuronal inputs by differential modulations of gamma-band phase-coherence. J. Neurosci..

[bb0225] Haegens S., Nacher V., Luna R., Romo R., Jensen O. (2011). Oscillations in the monkey sensorimotor network influence discrimination performance by rhythmical inhibition of neuronal spiking. Proc. Natl. Acad. Sci..

[bb0230] Haeusler S., Maass W. (2007). A statistical analysis of information-processing properties of lamina-specific cortical microcircuit models. Cereb. Cortex.

[bb0235] Haider B., Duque A., Hasenstaub A.R., McCormick D.A. (2006). Neocortical network activity in vivo is generated through a dynamic balance of excitation and inhibition. J. Neurosci..

[bb0240] Hupé J.M., James A.C., Payne B.R., Lomber S.G., Girard P., Bullier J. (1998). Cortical feedback improves discrimination between figure and background by V1, V2 and V3 neurons. Nature.

[bb0245] Jansen B.H., Rit V.G. (1995). Electroencephalogram and visual evoked potential generation in a mathematical model of coupled cortical columns. Biol. Cybern..

[bb0250] Jensen O., Mazaheri A. (2010). Shaping functional architecture by oscillatory alpha activity: gating by inhibition. Front. Hum. Neurosci..

[bb0255] Kaliukhovich D.A., Vogels R. (2012). Stimulus repetition affects both strength and synchrony of macaque inferior temporal cortical activity. J. Neurophysiol..

[bb0260] Lachaux J.P., Rodriguez E., Martinerie J., Varela F.J. (1999). Measuring phase synchrony in brain signals. Hum. Brain Mapp..

[bb0265] Larkman A.U. (1991). Dendritic morphology of pyramidal neurones of the visual cortex of the rat: III. Spine distributions. J. Comp. Neurol..

[bb0270] Litvak V., Eusebio A., Jha A., Oostenveld R., Barnes G., Foltynie T., Limousin P., Zrinzo L., Hariz M.I., Friston K., Brown P. (2012). Movement-related changes in local and long-range synchronization in Parkinson's disease revealed by simultaneous magnetoencephalography and intracranial recordings. J. Neurosci..

[bb0275] Livingstone M.S. (1996). Oscillatory firing and interneuronal correlations in squirrel monkey striate cortex. J. Neurophysiol..

[bb0280] Maier A., Adams G.K., Aura C., Leopold D.A. (2010). Distinct superficial and deep laminar domains of activity in the visual cortex during rest and stimulation. Front. Syst. Neurosci..

[bb0285] Markov N.T., Ercsey-Ravasz M.M., Ribeiro Gomes A.R., Lamy C., Magrou L., Vezoli J., Misery P., Falchier A., Quilodran R., Gariel M.A., Sallet J., Gamanut R., Huissoud C., Clavagnier S., Giroud P., Sappey-Marinier D., Barone P., Dehay C., Toroczkai Z., Knoblauch K., Van Essen D.C., Kennedy H. (2012). A weighted and directed interareal connectivity matrix for macaque cerebral cortex. Cereb. Cortex.

[bb0290] Markov N.T., Vezoli J., Chameau P., Falchier A., Quilodran R., Huissoud C., Lamy C., Misery P., Giroud P., Ullman S., Barone P., Dehay C., Knoblauch K., Kennedy H. (2013). The anatomy of hierarchy: feedforward and feedback pathways in macaque visual cortex. J. Comp. Neurol..

[bb0295] Melzer S., Michael M., Caputi A., Eliava M., Fuchs E.C., Whittington M.A., Monyer H. (2012). Long-range-projecting GABAergic neurons modulate inhibition in hippocampus and entorhinal cortex. Science.

[bb0300] Meyer H.S., Schwarz D., Wimmer V.C., Schmitt A.C., Kerr J.N.D., Sakmann B., Helmstaedter M. (2011). Inhibitory interneurons in a cortical column form hot zones of inhibition in layers 2 and 5A. Proc. Natl. Acad. Sci..

[bb0310] Moran R.J., Stephan K.E., Kiebel S.J., Rombach N., O'Connor W.T., Murphy K.J., Reilly R.B., Friston K.J. (2008). Bayesian estimation of synaptic physiology from the spectral responses of neural masses. NeuroImage.

[bb0315] Moran R.J., Symmonds M., Stephan K.E., Friston K.J., Dolan R.J. (2011). An in vivo assay of synaptic function mediating human cognition. Curr. Biol..

[bb0305] Moran R.J., Campo P., Symmonds M., Stephan K.E., Dolan R.J., Friston K.J. (2013). Free energy, precision and learning: the role of cholinergic neuromodulation. J. Neurosci..

[bb0320] Nalatore H., Ding M., Rangarajan G. (2007). Mitigating the effects of measurement noise on Granger causality. Phys. Rev. E Stat. Nonlinear Soft Matter Phys..

[bb0325] Nolte G., Bai O., Wheaton L., Mari Z., Vorbach S., Hallett M. (2004). Identifying true brain interaction from EEG data using the imaginary part of coherency. Clin. Neurophysiol..

[bb0330] Nolte G., Ziehe A., Nikulin V.V., Schlögl A., Krämer N., Brismar T., Müller K.-R. (2008). Robustly estimating the flow direction of information in complex physical systems. Phys. Rev. Lett..

[bb0335] Olsen S.R., Bortone D.S., Adesnik H., Scanziani M. (2012). Gain control by layer six in cortical circuits of vision. Nature.

[bb0340] Pinotsis D.A., Brunet N., Bastos A., Bosman C.A., Litvak V., Fries P., Friston K.J. (2014). Contrast gain control and horizontal interactions in V1: a DCM study. NeuroImage.

[bb0345] Rao R.P., Ballard D.H. (1999). Predictive coding in the visual cortex: a functional interpretation of some extra-classical receptive-field effects. Nat. Neurosci..

[bb0350] Rockland K.S., Pandya D.N. (1979). Laminar origins and terminations of cortical connections of the occipital lobe in the rhesus monkey. Brain Res..

[bb0355] Roopun A.K., Middleton S.J., Cunningham M.O., LeBeau F.E., Bibbig A., Whittington M.A., Traub R.D. (2006). A beta2-frequency (20–30 Hz) oscillation in nonsynaptic networks of somatosensory cortex. Proc. Natl. Acad. Sci..

[bb9000] Rubehn B., Bosman C., Oostenveld R., Fries P., Stieglitz T. (2009). A MEMS-based flexible multichannel ECoG-electrode array. J. Neural Eng..

[bb0360] Saleem K.S., Logothetis N.K. (2007). A combined MRI and histology atlas of the rhesus monkey brain in stereotaxic coordinates.

[bb0365] Sherman S.M., Guillery R. (1998). On the actions that one nerve cell can have on another: distinguishing “drivers” from “modulators.”. Proc. Natl. Acad. Sci. U. S. A..

[bb0370] Sherman S.M., Guillery R.W. (2011). Distinct functions for direct and transthalamic corticocortical connections. J. Neurophysiol..

[bb0375] Shipp S. (2007). Structure and function of the cerebral cortex. Science.

[bb0380] Shlosberg D., Amitai Y., Azouz R. (2006). Time-dependent, layer-specific modulation of sensory responses mediated by neocortical layer 1. J. Neurophysiol..

[bb0385] Smith M.A., Jia X., Zandvakili A., Kohn A. (2013). Laminar dependence of neuronal correlations in visual cortex. J. Neurophysiol..

[bb0390] Summerfield C., Trittschuh E.H., Monti J.M., Mesulam M.-M., Egner T. (2008). Neural repetition suppression reflects fulfilled perceptual expectations. Nat. Neurosci..

[bb0395] Summerfield C., Wyart V., Johnen V.M., de Gardelle V. (2011). Human scalp electroencephalography reveals that repetition suppression varies with expectation. Front. Hum. Neurosci..

[bb0400] Todorovic A., van Ede F., Maris E., de Lange F.P. (2011). Prior expectation mediates neural adaptation to repeated sounds in the auditory cortex: an MEG study. J. Neurosci..

[bb0405] Van Kerkoerle T., Self M.W., Dagnino B., Gariel-Mathis M.-A., Poort J., van der Togt C., Roelfsema P.R. (2014). Alpha and gamma oscillations characterize feedback and feedforward processing in monkey visual cortex. Proc. Natl. Acad. Sci. U. S. A..

[bb0410] Vezoli J. (2004). Quantitative analysis of connectivity in the visual cortex: extracting function from structure. Neuroscientist.

[bb0415] Vinck M., Huurdeman L., Bosman C., Fries P., Battaglia F., Pennartz C., Tiesinga P. (2015). How to detect the Granger-causal flow direction in the presence of additive noise?. NeuroImage.

[bb0420] Von Stein A., Chiang C., König P. (2000). Top-down processing mediated by interareal synchronization. Proc. Natl. Acad. Sci..

[bb0425] Wang X.-J. (2010). Neurophysiological and computational principles of cortical rhythms in cognition. Physiol. Rev..

[bb0430] Watanabe H., Sato M.-A., Suzuki T., Nambu A., Nishimura Y., Kawato M., Isa T. (2012). Reconstruction of movement-related intracortical activity from micro-electrocorticogram array signals in monkey primary motor cortex. J. Neural Eng..

[bb0435] Xing D., Shen Y., Burns S., Yeh C.-I., Shapley R., Li W. (2012). Stochastic generation of gamma-band activity in primary visual cortex of awake and anesthetized monkeys. J. Neurosci..

